# The Effects of Dietary Supplementation of Chestnut Tannic Acid on the Growth Performance, Gut Morphology and Microbiota of Weaned Piglets

**DOI:** 10.3390/metabo15070477

**Published:** 2025-07-15

**Authors:** Jinzhou Zhang, Yuting Zhang, Yuya Wang, Yanwei Li, Dongyang Liu, Hongbing Xie, Yongqiang Wang, Meinan Chang, Liping Guo, Zhiguo Miao

**Affiliations:** 1 College of Animal Science and Veterinary Medicine, Henan Institute of Science and Technology, Xinxiang 453003, China; zhangjz69@hist.edu.cn (J.Z.); zyt1961285035@163.com (Y.Z.); wang2246421897@163.com (Y.W.); lyw080805@163.com (Y.L.); liudy97@126.com (D.L.); xhb9607@126.com (H.X.); netwyq@163.com (Y.W.); meinan5176@126.com (M.C.); 2 School of Food Science, Henan Institute of Science and Technology, Xinxiang 453003, China; lipingguo1982@126.com

**Keywords:** growth performance, intestinal health, gut microbiota, piglets, tannic acid

## Abstract

**Background/Objectives**: This study investigated the effects of chestnut tannic acid (TA) on the growth performance, the expression of tight junction proteins and the composition of the gut microbiota of weaned piglets, which could provide novel insights into the application of TA in swine production. **Methods**: In a 42-day trial, 180 healthy, 21-day-old Duroc × Landrace × Yorkshire piglets were randomly assigned to a Control group and four treatment groups (TA1–4), fed commercial diets supplemented with 0, 0.06%, 0.12%, 0.18% or 0.24% TA. Each group had six replicates of six pigs each. **Results**: The average daily gain in all TA groups, the jejunal and ileal villus height and the villus height-to-crypt depth ratio in the TA3 and TA4 groups were markedly increased (*p* < 0.05). The mRNA levels of *MUC2* and *ZO-1* were upregulated in the TA3 group, as were those of *MUC4* in the jejunum and ileum and *claudin* in the duodenum and ileum; glutathione peroxidase and total antioxidant capacity were upregulated in the duodenum and jejunum in the TA3 group, and total superoxide dismutase was increased in all the TA2 groups (*p* < 0.05). Conversely, the malondialdehyde significantly decreased in all the TA groups (*p* < 0.05). TA supplementation improved the alpha diversity of the intestinal microflora and augmented probiotic abundance while reducing that of pathogenic bacteria. The contents of acetic, isobutyric, valeric, isovaleric, hexanoic and propionic acids, as well as total short-chain fatty acids (SCFA), were higher in the TA2 and TA3 groups (*p* < 0.05). **Conclusions**: TA inclusion in piglet diets improved the intestinal environment by upregulating the antioxidant enzymes, improving intestinal morphology and promoting probiotic growth and SCFA production while reducing pathogenic bacterial abundance, consequently enhancing the gut barrier and the growth of weaned piglets.

## 1. Introduction

During weaning, the immune system of piglets is still underdeveloped, and their gut microbiota are imbalanced. Consequently, changes in feed traits and the environment can lead to a decrease in feed intake, impaired digestion and absorption, diarrhea and even death, which seriously affects the economic benefits of pig farms [1]. Farmers have traditionally added antibiotics to the feed of piglets to mitigate weaning stress; however, the long-term overuse of antibiotics has led to growing problems, such as the presence of drug residues in livestock products and bacterial resistance to antibiotics [2]. This underscores the need to find alternative environmentally friendly feed additives to replace antibiotics and alleviate weaning stress in piglets.

Tannins comprise a class of water-soluble polyphenolic compounds widely distributed in different tissues of plants, including the leaves, roots, fruit and epidermis [3]. The structure and content of tannins differ according to the type of plant, the growth phase of the plant and its environment, reflecting its multiple biological functions [4]. Based on their characteristics, tannins are used for a wide range of applications, especially in leather manufacturing [5]. Moreover, studies have shown that tannins have antioxidant, antibacterial, antiviral, anti-inflammatory, antiallergic and vasodilatory properties [6].

Chestnut tannic acid (TA) is mainly obtained from the European chestnut tree (Castanea sativa Mill; CAS number: 1401-55-4). One study demonstrated that TA supplementation affected the intestinal microbiota and short-chain fatty acid (SCFA) production of patients presenting with the diarrheal subtype of irritable bowel syndrome [7]. Liu et al. found that dietary TA supplementation improved the gut morphology and mucosal antioxidative capacity, digestive enzyme activities and volatile fatty acid composition in weaned piglets [8]. Another study showed that the dietary addition of 2 g/kg TA mitigated the reactive oxygen species-mediated damage to gut epithelial cells caused by heat stress in broilers [9]. Nuamah et al. found that the dietary addition of tannin improved the growth performance of weaned piglets by decreasing the feed conversion ratio and increasing the final body weight, as well as increased the serum immune indices and antioxidant capacity using a meta-analysis [10]. Moreover, recent studies have indicated that TA has potential prebiotic properties and can improve animal growth traits and health [11,12]. Nevertheless, the mechanism by which TA modulates the intestinal mucosal barrier and the gut microbiota remains ill-defined.

In this study, we hypothesized that TA could improve intestinal health in weaning piglets by influencing the expression of tight junction proteins and the composition of the gut microbiota. To test this possibility, we evaluated the effects of TA on growth traits, apparent digestibility of nutrients, intestinal morphology, tight junctional protein expression, the gut microbiota and the concentration of SCFAs in weaned piglets. Our findings provide novel insights into the application of chestnut TA in swine production.

## 2. Materials and Methods

### 2.1. Animals and Experimental Design

The animals and procedures were approved by the Animal Protection and Utilization Committee of the Henan Institute of Science and Technology, Xinxiang, China (Approval number: LLSC2024063). Tannic acid (70% content), extracted from chestnut wood, was purchased from Silvateam (Via Torre, S. Michele Mondovì, Cuneo, Italy). All animal-related procedures were approved by the Animal Protection and Utilization Committee of Henan Institute of Science and Technology, Xinxiang, China (Approval number 2023 HIST041). The animal experiments were performed at Xinxiang Lvfengyuan Agricultural Development Co., Ltd. (Xinxiang, China). A total of 180 healthy Duroc × Landrace × Yorkshire weaned piglets (average weight: 6.2 ± 0.13 kg; weanling age: 21 days; male and female in half) were provided by Xinxiang Lvfengyuan Agricultural Development Co., Ltd. and randomly divided into 5 groups, namely a Control group, fed a commercially available diet, and four TA treatment groups (TA1–4), fed the same diet but supplemented with 0.06%, 0.12%, 0.18% or 0.24% TA. Each group contained six replicates, with six pigs per replicate (sex-balanced). The dosage of TA added was selected based on previous studies [11,12]. The trial lasted for 49 days, including a 7-day pre-trial period. The basic diet was designed to meet the nutritional needs of weaned piglets according to National Research Council guidelines [13]. The ingredients and nutritional levels are listed in Supplementary Table S1. Throughout the trial, all pigs were reared in the same pigsty with good ventilation and under an appropriate temperature (23 ± 2 °C) and relative humidity (40–60%). All piglets had free access to feed and water. Management, disinfection, vaccination and deworming were performed according to the procedures of the pig farm. At the start and finish of the trial, all piglets were weighed, while their feed consumption was repeatedly recorded. Subsequently, the average daily gain (ADG), average daily feed intake (ADFI) and feed-to-gain ratio (F/G) of the piglets were determined. The incidence and severity of diarrhea in piglets were recorded throughout the experiment and were evaluated through fecal consistency scores at 08:00 and 17:00 h daily according to the criteria in Supplementary Table S2.

### 2.2. Sample Collection

At the end of the trial, the piglets were fasted for 12 h while retaining free access to water. Two piglets (one male and one female) per replicate, with a body weight close to the average for their replicate, were selected for euthanasia according to the procedures of the slaughterhouse. After slaughter, the abdominal cavity of the animals was immediately opened, and the intestine was separated. A 10 cm section was collected from the middle segment of the duodenum, jejunum and ileum. Each intestinal segment was cut longitudinally, and the intestinal contents were rinsed with normal pre-cooled, sterile saline. Approximately 2 cm of each segment were then sampled, fixed in a 4% paraformaldehyde solution and kept at room temperature for morphological analysis. After scraping with a sterilized slide on an ice bath, the gut mucosa of the remaining samples was placed in RNA-free tubes, immediately frozen in liquid nitrogen and stored at −80 °C for the determination of gene expression levels and antioxidant properties.

After excising the cecum, the contents of the middle part were dispensed into 1.5 mL sterile tubes and kept at −80 °C for analysis of cecal microbiota and SCFA contents.

### 2.3. Analysis of Intestinal Morphology

The jejunal and ileal samples previously fixed in 4% paraformaldehyde were washed overnight with distilled water, dehydrated with ethanol, embedded in paraffin, sliced and stained with hematoxylin and eosin. Finally, the villus height (VH) and crypt depth (CD) were observed and imaged with a light microscope (TE2000-S, Nikon, Tokyo, Japan), and the villus height-to-crypt depth (V/C) ratio was determined.

### 2.4. Analysis of Antioxidant Properties

After thawing, the intestinal mucosal samples were added to a pre-cooled normal saline (V: W = 9:1), homogenized in an ice-water bath for 2 min and then centrifuged at 4 °C and 3500 r/min for 10 min (Avanti J-E, Beckman Coulter, Inc., Brea, CA, USA). The supernatant was collected and used to measure the antioxidant parameters (malondialdehyde (MDA) contents, total superoxide dismutase (T-SOD) and glutathione peroxidase (GSH-Px) activities and total antioxidant capacity (T-AOC)) using the respective ELISA kits (Nanjing Jiancheng Institute of Biotechnology Co., Ltd., Nanjing, China).

### 2.5. Quantitative Real-Time PCR (qPCR)

Frozen samples of the intestinal mucosa were homogenized in 1 mL of Trizol reagent (TaKaRa, Beijing, China) for total RNA extraction. RNA concentration and quality were analyzed using a NanoDropND-1000 spectrophotometer (Thermo Fisher Scientific, Waltham, MA, USA). Reverse transcription was performed with the PrimeScript RT reagent Kit with gDNA Eraser (TaKaRa, Beijing, China) following the kit instructions. The relative levels of mRNAs of gut mucosal barrier-related genes were detected by qPCR and determined using the 2^−ΔΔCt^ method. β-Actin served as the housekeeping gene. The sequences of the primers used for qPCR are presented in Supplementary Table S3, and their validation results were shown in Supplementary Figure S1.

### 2.6. Cecal Microbiota Assay

Total cecal bacterial DNA was extracted using the Omega Soil DNA Kit (v.D5625-01) (Omega Bio Tek, Norcross, GA, USA). Total DNA concentrations were evaluated using a NanoDropND-1000 spectrophotometer (Thermo Fisher Science). The V3–V4 hypervariable region of the bacterial 16S rRNA gene was PCR-amplified with forward primer 338F (5′-ACTCCTACGGGAGGCAGCA-3′) and reverse primer 806R (5′-GACTACHVGGGTWTCTAAT-3′). The obtained DNA fragments were sequenced on the Illumina NovaSeq6000 platform by Shanghai Paiseno Biotechnology Co., Ltd. (Shanghai, China).

The obtained raw tags were spliced and filtered to obtain clean tags. USEARCH (v.7.0.1090, https://drive5.com/usearch/(accessed on 15 September 2024) was used for clustering at ≥97% similarity, yielding representative operational taxonomic unit (OTU) sequences. The OTUs were compared to the Greengene database using RDP classifier (v.2.2) [14] for species annotation. D Species diversity and evenness were evaluated based on the alpha diversity index, and the *t*-test was used to assess the significance of inter-group differences using QIIME2 (version 2019.4) [15]. Beta diversity analysis was employed to evaluate the differences in species composition within and between groups with R packages Vegan (version v.3.2.0) [16]. Principal component analysis (PCA) based on OTU physical distances and principal coordinate analysis (PCoA) according to unweighted UniFrac distances were performed to visualize sample distribution. The significance of differences in microbial community structure between groups was evaluated using PERMANOVA (Adonis/PERMANOVA analysis). Linear discriminant analysis (LDA) effects size (LefSe) was used to identify marker species in each group using the Mann–Whitney test (LDA score ≥ 2) [17].

### 2.7. Assay for SCFAs in Cecal Contents

The SCFAs in cecal contents were separated and detected with an Agilent 1260 Infinity HPLC system (Agilent Technologies, Santa Clara, CA, USA) coupled to a SCIEX QTRAP 4500 mass spectrometer (Applied Biosystems, Framingham, MA, USA). Helium was used as the carrier gas, and the flow rate was 1 mL/min. The temperature program was set as follows: the initial temperature was 90 °C, ramping up to 100 °C at 20 °C/min, to 150 °C at 5 °C/min; then to 250 °C at 20 °C/min and held for 2 min [17]. The values of SCFAs were indicated by μg/g dry matter (DM).

### 2.8. Statistical Analysis

The trial data were analyzed with SPSS 22.0 software. One-way ANOVA was performed to determine the effects of the different concentrations of TA in the feed on a variety of indicators in the weaned piglets. Duncan’s method was used for multiple comparisons. All data are presented as means ± SEM, with *p* < 0.05 indicating significant differences.

## 3. Results

### 3.1. Growth Performance

As shown in Table 1, no differences in the initial body weight or F/G ratio were found among the groups (*p* > 0.05). Compared with the Control group, the final body weight and ADG of the weaned piglets were higher in all TA groups, while the ADFI was higher in the TA2, TA3 and TA4 groups (*p* < 0.05). Furthermore, the diarrhea index scores in the TA2 and TA3 groups were markedly reduced compared with those in the Control group (*p* < 0.05). The highest ADG, F/G and ADFI and lowest diarrhea index were found in the TA3 group. Interestingly, the effect on ADG of the TA4 group was lower with the TA3 group (*p* < 0.05).

### 3.2. Intestinal Morphology and Barrier Function

As shown in Table 2, compared with the Control group, the VH and V/C ratio was significantly increased in the jejunum of the TA2, TA3 and TA4 groups, and the ileum of all TA groups (*p* < 0.05), with the most prominent effect recorded in the TA3 group. No differences in the jejunal or ileal CD were detected among the groups (*p* > 0.05).

**Figure 1:**
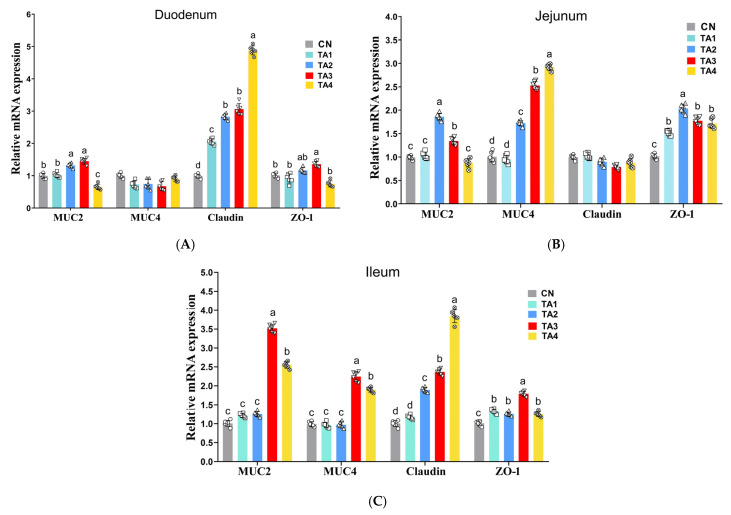
The expression level of genes related to the intestinal barrier in weaned piglets. (**A**) Duodenum; (**B**) jejunum; (**C**) ileum. *MUC2*, mucin 2; *MUC4*, mucin 4; *ZO-1*, zonula occludens-1; CN, Control; TA1–4, the groups fed the same diet supplemented with 0.06%, 0.12%, 0.18% or 0.24% tannic acid. For each gene, columns labeled with different lowercase letters differ significantly from each other (*p* < 0.05).

As shown in Figure 1A, compared with the Control group, the mRNA expression levels of *MUC2* were significantly upregulated in the TA2 and TA3 groups; similarly, *claudin* mRNA levels were increased in all the TA treatment groups, as were those of *ZO-1* in the TA3 group in the duodenum (*p* < 0.05). Additionally, in the jejunum (Figure 1B), compared with the Control group, the mRNA level of *MUC2* was significantly upregulated in the TA2 and TA3 groups, and that of *MUC4* was notably increased in the TA2, TA3 and TA4 groups, while *ZO-1* transcript levels were markedly higher in all the TA treatment groups (*p* < 0.05). In the ileum (Figure 1C), the *MUC2* and *MUC4* mRNA expression levels were considerably upregulated in the TA3 and TA4 groups; *claudin* mRNA levels were significantly increased in the TA2, TA3 and TA4 groups; and *ZO-1* expression levels were markedly higher in piglets of all the TA groups than in those of the Control group (*p* < 0.05). However, no differences in the mRNA levels of *MUC4* in the duodenum or *claudin* in the jejunum were observed among the groups (*p* > 0.05).

### 3.3. Antioxidant Capacity

As shown in Table 3, compared with the Control group, the MDA tended to decrease in all the TA groups (*p* < 0.05); T-SOD activities were higher in the TA3 group (*p* < 0.05); GSH-Px and the T-AOC was significantly increased in the TA3 groups in the duodenum and jejunum (*p* < 0.05). Meanwhile, TA3 consistently enhanced T-SOD, GSH-Px (except Ileum) and T-AOC (except Ileum), and reduced MDA (*p* > 0.05). However, no differences in GSH-Px or T-AOC in the ileum were found among the groups (*p* > 0.05).

### 3.4. Cecal Microbiota

#### 3.4.1. Analysis of Cecal Floral Abundance

**Figure 2:**
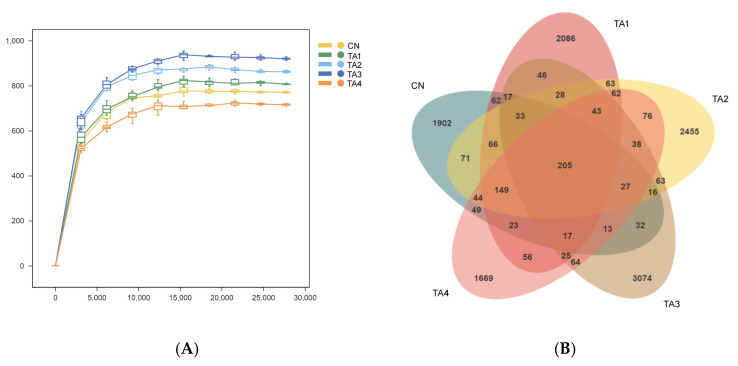
Analysis of cecal flora abundance in weaned piglets. (**A**) Species accumulation curves; (**B**) Venn diagram of the OTU analysis. CN, Control; TA1–4, the groups fed the same diet supplemented with 0.06%, 0.12%, 0.18% or 0.24% tannic acid.

As shown in Figure 2A, the species accumulation curve gradually flattened, indicating that the sequencing depth was sufficient to capture nearly all the species in the samples and adequately reflected species diversity. Additionally, 205 OTUs were shared among the groups, while 1902, 2086, 2455, 3074 and 1669 OTUs were unique to the Control, TA1, TA2, TA3 and TA4 groups, respectively (Figure 2B).

#### 3.4.2. Analysis of the Alpha Diversity in the Cecal Flora

As shown in Table 4, compared with the control group, the Chao1 index was significantly higher in all the TA supplementation groups. Furthermore, the number of observed species was higher in the TA2 and TA3 groups than in the Control group, as was Faith’s-PD index in the TA3 group (*p* < 0.05). However, no differences in the Pielou, Shannon, Simpson or Good’s coverage indices were detected among the groups (*p* > 0.05).

#### 3.4.3. Analysis of Beta Diversity in the Cecal Flora

**Figure 3:**
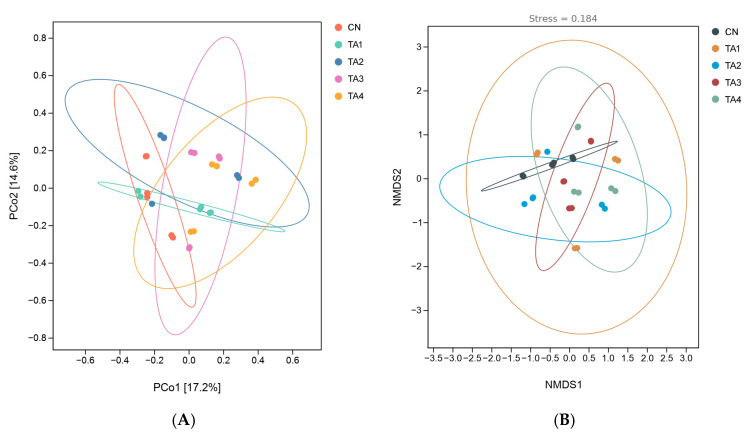
Beta diversity analysis of the cecal flora in weaned piglets. (**A**) Principal coordinates analysis; (**B**) non-metric multi-dimensional scaling analysis. PCo1, principal component 1; PCo2, principal component 2; NMDS1, non-metric multi-dimensional scaling analysis 1; NMDS2, non-metric multi-dimensional scaling analysis 2; CN, Control; TA1–4, the groups fed the same diet supplemented with 0.06%, 0.12%, 0.18% or 0.24% tannic acid.

As depicted in Figure 3A, principal component (PC) 1 and PC2 contributed 17.2% and 14.6% to the observed variance, respectively. Non-metric multidimensional scaling (NMDS) was employed to analyze species information in the samples in the form of dots, with the distance between the dots reflecting the degree of variation within and between groups (Figure 3B). A stress value of 0.18 was obtained in the NMDS analysis, which was less than the threshold of 0.20, indicating that NMDS accurately represented the degree of variation between samples.

#### 3.4.4. Analysis of Cecal Flora Composition

The major taxa of the cecal microbiota of the piglets at the phylum level are shown in Table 5. Firmicutes and Bacteroidetes were the dominant phyla, accounting for more than 92% of the classified phyla. Compared to the Control group, the relative abundances of Firmicutes were higher in all the TA groups, while those of Bacteroidetes and Proteobacteria was lower in the TA2, TA3 and TA4 groups (*p* < 0.05). No differences in the relative abundances of Actinobacteria or Tenericutes were found among the groups (*p* > 0.05).

At the family level, the top six core cecal flora in the piglets were, in order of abundance, *Lactobacillaceae*, *Prevotellaceae*, *Ruminococcaceae*, *Lachnospiraceae*, *Clostridiaceae* and *Streptococcaceae*. The relative abundance of *Lactobacillaceae* was significantly higher in the TA2 and TA3 groups than in the Control group, as was that of *Ruminococcaceae* in the TA1, TA3 and TA4 groups and that of *Clostridiaceae* in all the TA groups. In contrast, the abundance of *Prevotellaceae* was notably lower in the TA2, TA3 and TA4 groups than in the Control group (*p* < 0.05). No differences in the relative abundance of *Lachnospiraceae* or *Streptococcaceae* were found among the groups (*p* > 0.05) (Table 6).

As shown in Table 7, at the genus level, the dominant cecal flora were *Lactobacillus*, *Prevotella*, *Gemmiger*, *Streptococcus*, *Blautia*, *SMB53*, *Roseburia* and *Faecalibacterium*, in order of abundance. There were no differences in the relative abundances of *Gemmiger*, *Streptococcus*, *Blautia* and *Roseburia* among the groups (*p* > 0.05). Additionally, the abundance of *Lactobacillus* was higher in the TA2 and TA3 groups than in the Control group, and a similar trend was observed for SMB53 in the TA3 and TA4 groups and *Faecalibacterium* in the TA1 and TA3 groups (*p* < 0.05). However, the prevalence of *Prevotella* was lower in piglets of the TA2, TA3 and TA4 groups than in those of the Control group (*p* < 0.05).

#### 3.4.5. LefSe Species Variance Analysis

**Figure 4:**
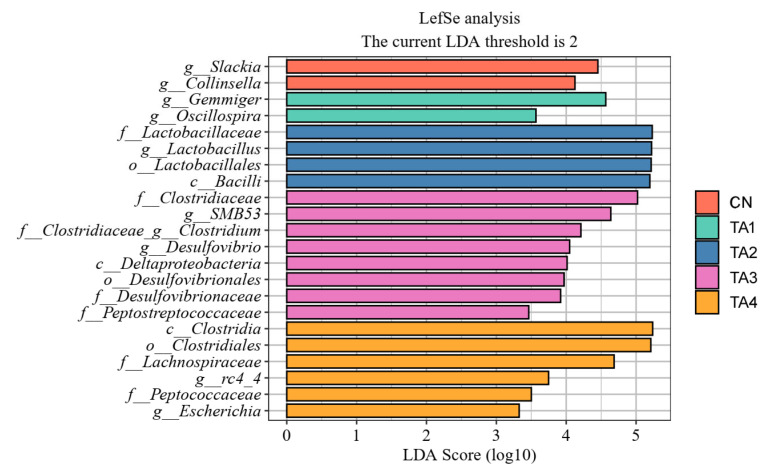
The LefSe species variance analysis in the cecal flora of weaned piglets. LefSe, linear discriminant analysis effects size; CN, Control; TA1–4, the groups fed the same diet supplemented with 0.06%, 0.12%, 0.18% or 0.24% tannic acid.

LefSe analysis revealed five distinct groups of significantly enriched biomarkers (LDA scores > 2.0, *p* < 0.05) in the cecal flora. As illustrated in Figure 4, the Control group exhibited the highest relative abundances of *Slackia* and *Gollinsella*. Meanwhile, *Gemmiger* and *Oscillospira* were most prevalent in the TA1 group; *Lactobacillaceae*, *Lactobacillus*, *Lactobacillales* and *Bacilli* dominated the TA2 group; the TA3 group showed the highest levels of *Clostridiaceae*, *SMB53*, *Clostridium*, *Desulfovibrio*, *Deliaproterobacteria*, *Desulfovibrionales*, *Desulfovibrionaceae* and *Peptostreptococaceae*; and *Clostridia*, *Clostridiales*, *Lachnospiraceae*, *rc4_4*, *Peptococcaceae* and *Escherichia* were most abundant in the TA4 group.

### 3.5. Analysis of SCFAs in Cecal Contents

The effects of TA on SCFA production in the cecum of the piglets are demonstrated in Table 8. The contents of acetic acid, isobutyric acid, valeric acid and total SCFAs were higher in the TA2 and TA3 groups than in the Control group, as were those of isovaleric acid and hexanoic acid in the TA2, TA3 and TA4 groups and propionic acid in the TA1, TA2 and TA3 groups (*p* < 0.05). However, no significant difference in the level of butyric acid was found among the groups (*p* > 0.05).

## 4. Discussion

Traditionally, dietary TA has been viewed as an antinutritional factor that negatively impacts animal feed intake and growth performance [18]. A study by Schiavone et al. showed that growth performance was significantly improved in broilers fed 0.2% natural extract of chestnut wood, with no adverse effects on organ health being detected [19]. In line with these reports, we observed that the dietary inclusion of 0.18% TA (TA3 group) significantly improved the ADG, while simultaneously decreasing diarrhea index scores in weaned piglets. However, some negative effects were noted with the addition of 0.24% TA (TA4 group) to the diet. This may be due to the ability of tannic acid to bind starch, proteins and digestive enzymes to form insoluble complexes, resulting in higher levels of tannic acid in diets that reduce palatability, nutrient digestibility and growth.

Villus height, crypt depth and their ratio are commonly used parameters for evaluating gut function and absorption status [20,21]. Zhao et al. found that the addition of 0.1% tannins to the diet of Hu sheep increased the VH in the jejunum and ileum and had a tendency to decrease CD in the ileum [22]. A separate study revealed that adding 0.2% TA to the diet decreased the CD and increased the V/C ratio in the duodenum, while 1.0% TA tended to reduce CD and VH in the ileum [12]. Our findings showed that supplementing the diet of weaned piglets with an appropriate amount of TA improved the VH and the V/C ratio and exerted a positive effect on the morphology of the gut mucosa. In turn, this enhanced the growth and intestinal health of the weaned piglets, with the best effect being achieved with the addition of 0.18% TA. Meanwhile, there was a dose-dependent effect of TA, with a reduced efficacy of TA4 (0.24%) indicating a dose-dependent threshold.

Tight junction proteins and mucins are essential components of the intestinal barrier, and a decrease in their expression can lead to increased intestinal permeability and inflammation [23]. Research has shown that early weaning in piglets is often accompanied by a reduction in tight junction protein levels, an increase in intestinal permeability and the occurrence of diarrhea [1]. Our findings suggested that TA supplementation in piglet diets upregulated the expression of *ZO-1*, *claudin*, *MUC2* and *MUC4* in the intestine of weaned piglets, implying that TA can alleviate weaning stress-induced damage in the gut barrier.

Weaning readily leads to excessive free radical production and oxidative stress in piglets [24]. The activities of T-SOD and GSH-Px, along with T-AOC and MDA levels, serve as important indicators for assessing antioxidant capacity [25]. Our findings showed that adding TA to the diet of weaned piglets improved GSH-Px activity and T-AOC in the duodenum and jejunum, increased T-SOD activity in the intestine and tended to lower MDA generation. Again, 0.18% was found to be the optimal TA supplementation level. These findings suggest that TA improves intestinal morphology and barrier integrity, at least in part by enhancing intestinal antioxidant capacity and reducing intestinal damage.

The gut microbiota and their metabolites are closely related to the physiological activities of animals, such as nutrition, metabolism and stress responses [26]. Accordingly, gut dysbiosis is considered to be a key factor responsible for post-weaning diarrhea and intestinal infections. In this study, alpha diversity analysis showed that dietary TA increased the Chao1 and Faith’s PD indexes, which implied that TA increased the diversity of the gut flora in the weaned piglets. In this study, Good’s coverage in each group exceeded 99%, thereby accurately reflecting the community composition of the fecal flora of piglets.

In this work, at the phylum level, we observed that the dominant cecal microbial communities in the piglets were Bacteroidetes and Firmicutes. Firmicutes can break down cellulose and degrade complex macromolecules, thereby improving the body’s immunity, digestion rate and resistance to pathogens. These effects are primarily mediated through the production of SCFAs, which modulate inflammation and serve as an energy source for gut epithelial cells. Bacteroidetes have high polysaccharide-degrading capacity, which contributes to enhanced host immunity and improved intestinal mucosal barrier function [27]. Here, we found that TA supplementation elevated the relative abundance of Firmicutes and the Firmicutes/Bacteroidetes ratio while concurrently reducing the relative abundance of Bacteroidetes. At the family level, the most abundant cecal microorganisms in the weaned piglets were *Lactobacillaceae*, followed by *Prevotellaceae* and *Ruminococcaceae*. Notably, TA addition increased the abundances of *Lactobacillaceae* and *Rumatobacteriaceae* and decreased that of *Prevotellaceae* in the piglet cecum. *Lactobacillaceae* are the prominent probiotic in the gut and can ameliorate intestinal permeability and barrier function by increasing occludin expression and suppressing the proliferation of harmful bacteria [28]. *Prevotellaceae* are involved in the regulation of glucose metabolism and were reported to be positively associated with gestational diabetes [29]. *Rumatococcaceae* are the main microorganisms responsible for transforming primary bile acids into secondary ones, thereby stabilizing the intestinal barrier and reducing gut inflammation [30]. At the genus level, we found that TA elevated the relative abundances of *Lactobacillus* and *SMB53* and reduced that of *Prevotella* in the cecal flora of the piglets. In pigs, *Lactobacillus* plays an important part in regulating immunity, sustaining homeostasis and health, aiding digestion and improving growth [25]. *SMB53*, a member of the *Clostridiaceae* family, can consume intestinal mucus and plant-derived sugars, suggesting that it is involved in sugar metabolism [31]. *Prevotella* is an opportunistic pathogen that not only ferments non-starch polysaccharides, yielding SCFAs, but also increases intestinal permeability, which leads to inflammation [32]. LefSe analyses showed that the cecal flora of the weaned piglets in the Control group was rich in *Slackia* and *Collinsella*, potentially explaining the occurrence of diarrhea in the piglets. *Slackia* abundance is notably elevated in chickens with low body weight, and these bacteria promote the deposition of body fat in animals [33]. The influence of *Collinsella* in glucose-lipid metabolism may be related to its involvement in the inflammatory response and the disruption of the gut mucosal barrier [34]. Piglets in the TA2 group had the highest abundances of *Lactobacillaceae*, *Lactobacillus*, *Lactobacillales* and *Bacilli*, while the greatest relative abundances of *Clostridiaceae*, *SMB53*, *Clostridium*, *Desulfovibrio*, *Deliaproterobacteria*, *Desulfovibrionales*, *Desulfovibrionaceae* and *Peptostreptococaceae* were recorded in the TA3 group. These results suggest that dietary TA supplementation improves gut health and growth in weaned piglets by increasing the abundance of probiotics and lowering that of pathogenic bacteria in the intestinal microbiota.

Short-chain fatty acids, the main metabolites generated by intestinal flora through the fermentation of dietary fiber and starch, are saturated fatty acids with a carbon chain of six or fewer carbon atoms. These include formic, acetic, propionic, butyric, valeric and hexanoic acids, along with their isomers. A previous has demonstrated that SCFAs can promote the growth and maturation of intestinal tissue, maintain the integrity of the gut barrier and mitigate gut inflammation [35]. In this work, we found that dietary TA supplementation led to an increase in the contents of acetic, propionic, isobutyric, hexanoic, valeric and isovaleric acids, as well as in the total SCFA content in the cecum of piglets, indicating that adding TA to the diet exerts a positive effect on the gut flora of weaned piglets. The best effect was seen with the 0.18% TA inclusion level. However, there was no significant change in the content of butyric acid, which was inconsistent with the previous report [8], probably due to the difference in the type and added amount of TA, and the specific reason needs to be further investigated. We speculate that dietary TA improves the intestinal environment in weaned piglets by elevating the production of SCFAs, thereby contributing to improved growth performance.

## 5. Conclusions

These findings suggest that the inclusion of TA in the diet of weaned piglets improves the intestinal environment by upregulating the antioxidant enzymes, improving intestinal morphology, increasing the abundance of probiotics and the production of SCFAs and reducing the abundance of pathogenic bacteria in the gut microbiota. Collectively, these effects strengthen the intestinal barrier and improve the growth performance of weaned piglets. The 0.18% TA in piglet diets is optimal for enhancing gut health and growth. This study provides novel insights into the application of TA as a feed additive in pig production.

## Figures and Tables

**Table 1:** The effect of tannic acid on the growth performance of weaned piglets.

Groups							
Item	CN	TA1	TA2	TA3	TA4	SEM	*p*-Value
Initial BW, kg	7.38	7.35	7.40	7.37	7.41	0.13	0.560
Final BW, kg	29.88 ^d^	30.58 ^c^	31.17 ^b^	32.83 ^a^	30.53 ^c^	0.35	0.031
ADFI, g	977.61 ^c^	988.09 ^bc^	1012.91 ^b^	1044.08 ^a^	1001.26 ^b^	21.78	0.023
ADG, g	535.71 ^c^	553.10 ^b^	565.95 ^b^	586.19 ^a^	550.48 ^b^	17.23	0.005
F/G	1.82	1.79	1.79	1.78	1.82	0.02	0.270
Diarrhea index	2.35 ^a^	2.23 ^ab^	2.10 ^b^	1.98 ^c^	2.19 ^ab^	0.04	0.002

ADFI, average daily feed intake; ADG, average daily gain; BW, body weight; F/G, feed-to-gain ratio; CN, Control; TA1–4, the groups fed the same diet supplemented with 0.06%, 0.12%, 0.18% or 0.24% tannic acid. In the same row, values labeled with different superscript lowercase letters differ significantly from each other (*p* < 0.05).

**Table 2:** The effect of tannic acid on jejunal and ileal morphology in weaned piglets.

Groups							
Item	CN	TA1	TA2	TA3	TA4	SEM	*p*-Value
**Jejunum**							
VH, μm	444.60 ^c^	441.76 ^c^	508.89 ^b^	581.19 ^a^	507.25 ^b^	15.73	<0.001
CD, μm	252.15	249.66	230.16	234.53	238.98	5.21	0.333
V/C	1.76 ^c^	1.77 ^c^	2.21 ^b^	2.48 ^a^	2.12 ^b^	0.09	0.002
**Ileum**							
VH, μm	452.10 ^c^	517.01 ^b^	531.79 ^b^	561.02 ^a^	512.92 ^b^	16.51	<0.001
CD, μm	320.31	335.20	344.34	329.43	328.04	7.91	0.311
V/C	1.41 ^c^	1.54 ^b^	1.54 ^b^	1.70 ^a^	1.56 ^b^	0.06	<0.001

CD, crypt depth; VH, villus height; V/C, villus height-to-crypt depth; CN, Control; TA1–4, the groups fed the same diet supplemented with 0.06%, 0.12%, 0.18% or 0.24% tannic acid. In the same row, values labeled with different superscript lowercase letters differ significantly from each other (*p* < 0.05).

**Table 3:** The effect of tannic acid on the antioxidant capacity of weaned piglets.

Groups							
Item	CN	TA1	TA2	TA3	TA4	SEM	*p*-Value
**Duodenum**							
MDA, nmol/mL	1.76 ^a^	1.13 ^b^	0.40 ^c^	0.30 ^c^	0.84 ^b^	0.15	<0.001
T-SOD, U/mL	89.41 ^b^	83.36 ^b^	91.49 ^b^	109.94 ^a^	90.32 ^b^	3.50	0.041
GSH-Px, U/mL	20.59 ^b^	22.23 ^b^	20.92 ^b^	28.16 ^a^	22.81 ^b^	0.81	0.001
T-AOC, nmol/mL	0.19 ^b^	0.18 ^b^	0.26 ^a^	0.28 ^a^	0.22 ^ab^	0.01	0.015
**Jejunum**							
MDA, nmol/mL	1.2 ^a^	0.77 ^b^	0.75 ^b^	0.50 ^c^	0.31 ^c^	0.08	<0.001
T-SOD, U/mL	72.36 ^c^	97.97 ^b^	87.64 ^bc^	129.09 ^a^	130.42 ^a^	6.47	<0.001
GSH-Px, U/mL	17.72 ^c^	18.17 ^c^	17.47 ^c^	39.92 ^a^	21.74 ^b^	2.31	<0.001
T-AOC, nmol/mL	0.13 ^c^	0.21 ^b^	0.26 ^ab^	0.32 ^a^	0.21 ^b^	0.02	0.024
**Ileum**							
MDA, nmol/mL	1.31 ^a^	1.23 ^a^	1.08 ^b^	0.55 ^c^	0.54 ^c^	0.13	0.042
T-SOD, U/mL	69.54 ^b^	67.62 ^b^	75.71 ^ab^	86.44 ^a^	69.33 ^b^	3.07	0.033
GSH-Px, U/mL	32.60	32.11	34.35	40.94	32.97	1.27	0.137
T-AOC, nmol/mL	0.20	0.18	0.20	0.25	0.20	0.01	0.163

GSH-Px, glutathione peroxidase; MDA, malondialdehyde; T-AOC, total antioxidant capacity; T-SOD, total superoxide dismutase; CN, Control; TA1–4, the groups fed the same diet supplemented with 0.06%, 0.12%, 0.18% or 0.24% tannic acid. In the same row, values labeled with different superscript lowercase letters differ significantly from each other (*p* < 0.05).

**Table 4:** Analysis of alpha diversity.

Groups							
Item	CN	TA1	TA2	TA3	TA4	SEM	*p*-Value
Chao1	770.76 ^d^	808.11 ^c^	862.15 ^b^	920.76 ^a^	716.13 ^e^	35.58	0.029
Pielou	0.67	0.66	0.65	0.71	0.65	0.01	0.206
Observed species	729.18 ^b^	747.03 ^b^	831.17 ^a^	859.65 ^a^	675.22 ^c^	33.99	0.430
Faith’s PD	48.79 ^b^	49.22 ^b^	54.91 ^ab^	60.83 ^a^	48.90 ^b^	1.55	0.031
Shannon	6.38	6.24	6.34	6.95	6.11	0.23	0.280
Simpson	0.95	0.93	0.90	0.97	0.94	0.04	0.058
Goods coverage	1.00	1.00	1.00	0.99	1.00	<0.01	0.069

CN, Control; TA1–4, the groups fed the same diet supplemented with 0.06%, 0.12%, 0.18% or 0.24% tannic acid. In the same line, values labelled with different superscript lowercase letters differ significantly from each other (*p* < 0.05).

**Table 5:** The abundances of cecal microbiota at the phylum level (*n* = 6, %).

Groups							
Item	CN	TA1	TA2	TA3	TA4	SEM	*p*-Value
Firmicutes	63.17 ^d^	68.46 ^c^	73.41 ^bc^	88.36 ^a^	79.66 ^b^	2.64	0.002
Bacteroidetes	32.07 ^a^	27.30 ^ab^	19.10 ^bc^	9.11 ^c^	16.33 ^bc^	2.62	0.014
Actinobacteria	0.04	0.03	0.05	0.02	0.01	0.01	0.447
Proteobacteria	3.82 ^a^	2.51 ^ab^	0.76 ^c^	0.52 ^c^	1.51 ^bc^	0.38	0.007
Tenericutes	0.54	0.19	0.22	0.21	0.66	0.11	0.082

CN, Control; TA1–4, the groups fed the same diet supplemented with 0.06%, 0.12%, 0.18% or 0.24% tannic acid. In the same line, values labelled with different superscript lowercase letters differ significantly from each other (*p* < 0.05).

**Table 6:** The abundances of cecal microbiota at the family level (*n* = 6, %).

Groups							
Item	CN	TA1	TA2	TA3	TA4	SEM	*p*-Value
*Lactobacillaceae*	33.85 ^c^	29.06 ^c^	55.25 ^a^	41.36 ^b^	23.35 ^d^	3.26	0.001
*Ruminococcaceae*	6.37 ^c^	16.88 ^a^	7.33 ^c^	12.09 ^b^	14.40 ^ab^	1.34	0.023
*Lachnospiraceae*	9.25	11.50	9.98	7.89	14.52	0.97	0.248
*Prevotellaceae*	23.76 ^a^	18.09 ^a^	3.89 ^c^	5.20 ^c^	11.04 ^b^	2.23	0.001
*Clostridiaceae*	0.44 ^d^	1.35 ^c^	2.80 ^c^	11.91 ^a^	7.32 ^b^	0.46	0.034
*Streptococcaceae*	4.02	0.57	2.38	3.18	2.41	0.54	0.379

CN, Control; TA1–4, the groups fed the same diet supplemented with 0.06%, 0.12%, 0.18% or 0.24% tannic acid. In the same line, values labelled with different superscript lowercase letters differ significantly from each other (*p* < 0.05).

**Table 7:** The abundances of cecal microbiota at the genus level (*n* = 6, %).

Groups							
Item	CN	TA1	TA2	TA3	TA4	SEM	*p*-Value
*Lactobacillus*	32.88 ^c^	29.93 ^c^	55.24 ^a^	41.35 ^b^	23.31 ^d^	3.20	<0.001
*Prevotella*	19.63 ^a^	21.34 ^a^	3.88 ^c^	5.19 ^c^	11.00 ^b^	2.48	0.041
*Gemmiger*	6.16	8.44	3.21	7.87	5.37	1.04	0.584
*Streptococcus*	4.43	0.48	2.36	3.16	2.38	0.61	0.381
*Blautia*	3.76	5.96	3.85	4.04	6.15	0.92	0.895
*SMB53*	0.03 ^c^	0.33 ^c^	0.90 ^c^	2.79 ^b^	3.70 ^a^	0.21	0.040
*Roseburia*	0.23	2.59	1.36	0.14	1.09	0.37	0.203
*Faecalibacterium*	0.83 ^b^	1.90 ^a^	0.65 ^b^	1.71 ^a^	0.96 ^b^	0.15	0.005

CN, Control; TA1–4, the groups fed the same diet supplemented with 0.06%, 0.12%, 0.18% or 0.24% tannic acid. In the same line, values labelled with different superscript lowercase letters differ significantly from each other (*p* < 0.05).

**Table 8:** Analysis of short-chain fatty acid contents in the cecal contents (*n* = 6, %).

Groups							
Item	CN	TA1	TA2	TA3	TA4	SEM	*p*-Value
Acetic acid	1966.1 ^b^	1972.9 ^b^	2251.2 ^a^	2267.0 ^a^	1892.5 ^b^	116.24	<0.001
Propionic acid	1340.0 ^c^	1539.0 ^b^	1532.4 ^b^	1651.4 ^a^	1367.8 ^c^	83.79	0.016
Isobutyric acid	41.1 ^b^	43.8 ^b^	74.9 ^a^	81.5 ^a^	49.5 ^b^	5.11	0.024
Butyric acid	1058.2	967.9	1014.5	938.7	927.5	71.02	0.325
Isovaleric acid	45.2 ^c^	50.4 ^c^	72.9 ^b^	91.4 ^a^	69.9 ^b^	7.64	0005
Valeric acid	202.1 ^b^	228.6 ^ab^	261.8 ^a^	241.2 ^a^	225.2 ^ab^	14.48	0.041
Hexanoic acid	8.0 ^b^	9.7 ^ab^	12.5 ^a^	11.4 ^a^	11.7 ^a^	1.13	0.003
Total_SCFA	4700.7 ^b^	4812.3 ^b^	5220.3 ^a^	5282.6 ^a^	4544.1 ^b^	235.65	<0.001

CN, Control; TA1–4, the groups fed the same diet supplemented with 0.06%, 0.12%, 0.18% or 0.24% tannic acid. In the same line, values labelled with different superscript lowercase letters differ significantly from each other (*p* > 0.05).

## Data Availability

Dataset available on request from the authors.

## Supplementary Materials

The following supporting information can be downloaded at: https://www.mdpi.com/article/10.3390/metabo15070477/s1. Table S1. The composition and nutritional level of the feed provided to the weaned piglets; Table S2. Fecal scoring criteria in weaned piglets; Table S3. The sequences of the primers used for qPCR; Figure S1. Their validation results of qPCR.
