# Supplementary material for: The Effects of Dietary Supplementation of Chestnut Tannic Acid on the Growth Performance, Gut Morphology and Microbiota of Weaned Piglets
Source: Metabolites. 2025 Jul 15;15(7):477. doi: 10.3390/metabo15070477 (PMC12300185; doi:10.3390/metabo15070477)
Supplement: Supplementary file 1 [file metabolites-15-00477-s001.zip › metabolites-3703105-supplementary.pdf]

Table S1. The composition and nutritional level of the feed provided to the weaned piglets.

Ingredient	Content (%)	Nutritional indicators[2]	Content
Puffed corn	50.00	DE kcal/kg	3494
Broken rice	10.00	ME kcal/kg	3362
Puffed soybean	8.00	CP %	19.03
Fish meal	2.00	Lys %	1.44
Soybean meal	8.00	SID Lys %	1.28
Fermented soybean meal	10.00	SID Thr %	0.75
Whey powder	6.00	SID Met %	0.36
Sucrose	2.00	SID Trp %	0.24
L-Lysine	0.60	SID Val %	0.62
Thr	0.20	SID Ile %	0.61
Met	0.10	SID Cys %	0.25
Try	0.10	SID Met+Cys %	0.61
Zinc oxide	0.10	Ca %	0.73
Phytase enzyme	0.01	TP %	0.57
Antioxidant/Ethoxyquinoline	0.02	AP %	0.37
Stone powder	1.00		
Dicalcium hydrogen phosphate	0.57		
Table salt	0.30		
Premix[1]	1.00		
Total	100		

[1] The premix provided the following per kg of diet: vitamin A, 9 570 IU; vitamin B_1, 2.50 mg; vitamin B_2, 7 mg; vitamin B_6, 5 mg; vitamin B_{12}, 0.05 mg; vitamin D_3, 2 500 IU; vitamin E, 30 mg; vitamin K_3, 2.50 mg; biotin, 0.15 mg; folic acid, 1.50 mg; D-pantothenic acid, 15 mg; nicotinic acid, 30 mg; Cu (as copper sulfate), 16 mg; Fe (as ferrous sulfate), 80 mg; Mn (as manganese sulfate), 70 mg; Zn (as zinc oxide), 80 mg.

[2] Nutrient indicators were calculated according to the Tables of Feed Composition and Nutritive Values in China (2015, twenty-sixth edition).

DE, digestible energy; ME, metabolic energy; CP, crude protein; SID, standardized ileal digestible; TP, total phosphorus; AP, available phosphorus.

Table S2. Fecal scoring criteria in weaned piglets.

Degree of diarrhea	Fecal status	Scores
Normal	Particles or strips	1
Mild	Soft, easy to shape	2
Moderate	Semi-liquid, unformed	3
Severe	Watery, unformed	4

Table S3. The sequences of the primers used for qPCR.

Gene	Sequence (5′-3′)	Amplicon sizes (bp)	Annealing temperatures (°C)	Accession no.
β-Actin	F: GGCACCACACCTTCTACAACGAG<br>R: TCATCTTCTCACGGTTGGCTTTGG	102	55	XM_021086047.1
MUC2	F: GACGACACCATCTACCTCACTCAG<br>R: TCTGTTCCACACGAGAGCAAGG	163	56	NM_002457.5
MUC4	F: CAGAAGACAGACAGCAAGACAAG<br>R: TGTGGAAGTGTGAGCAGTGGAAG	184	58	XM_021068272.1
ZO-1	F: CCTGAGTTTGATAGTGGCGTTGAC<br>R: ACGGTGTGACCATCCTCATCTTC	146	55	XM_021098856.1
Claudin	F: ACCGCCCACAATGTCATCCG<br>R: GCACCCATCTCCCGCTTCTG	171	56	NM_001161637.1

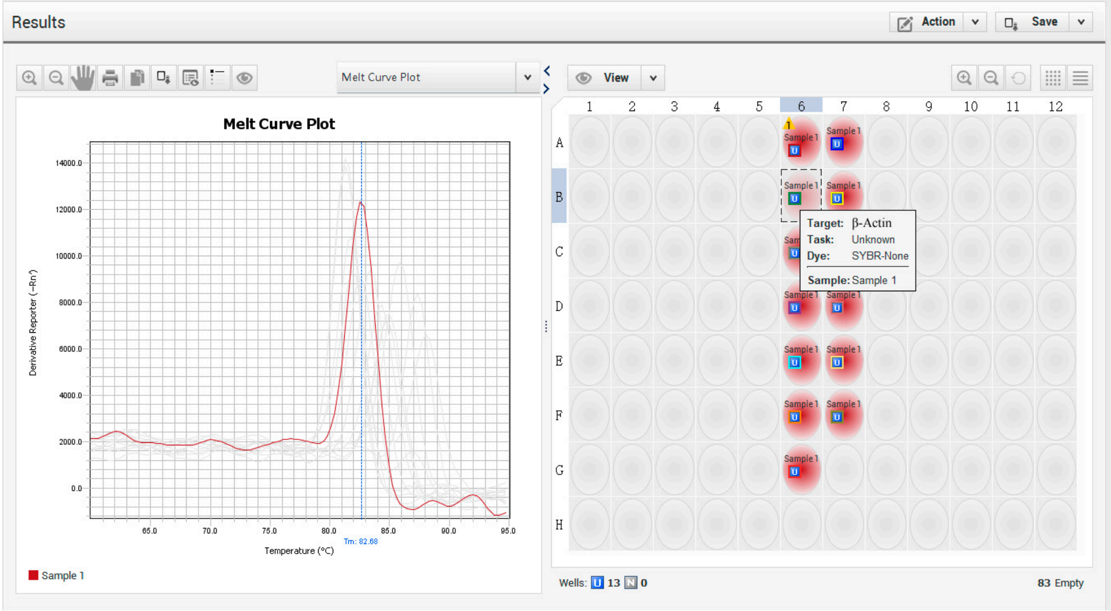

(A) *β-Actin*

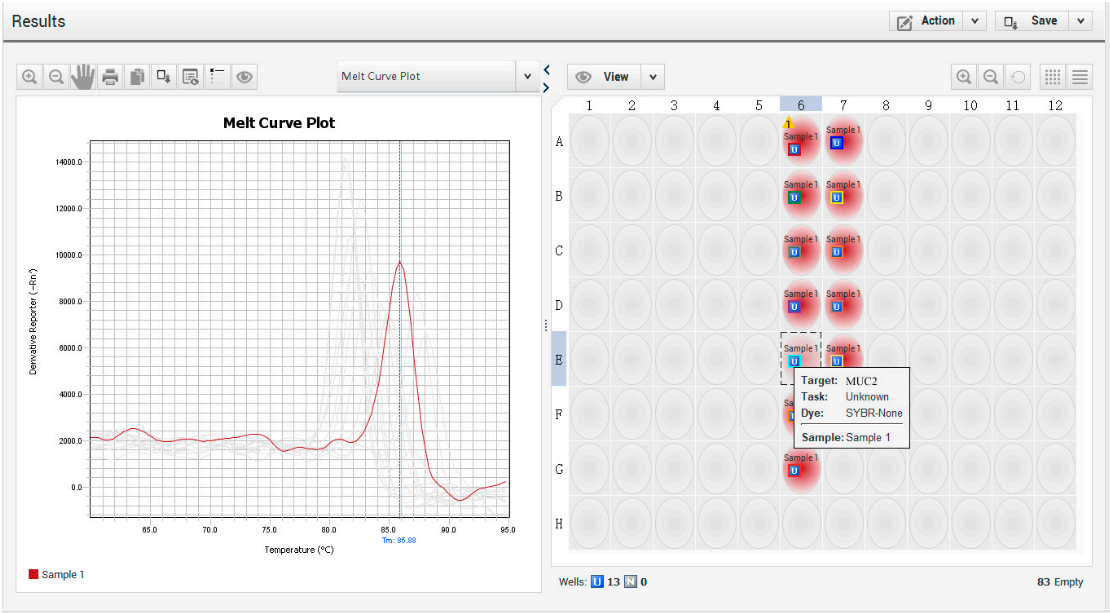

(B) *MUC2*

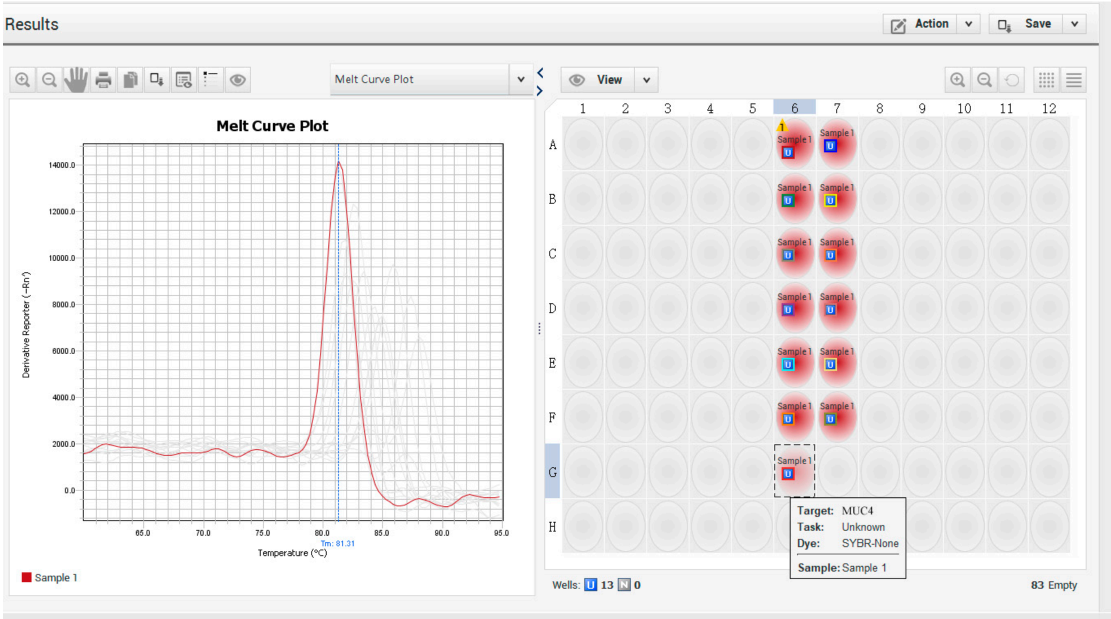
Results
Action
Save
Melt Curve Plot
View
Melt Curve Plot
Derivative Reporter (−Rn')
Temperature (°C)
Tm: 81.31
Sample 1
Target: MUC4
Task: Unknown
Dye: SYBR-None
Sample: Sample 1
Wells: U 13 N 0
83 Empty

(C) *MUC4*

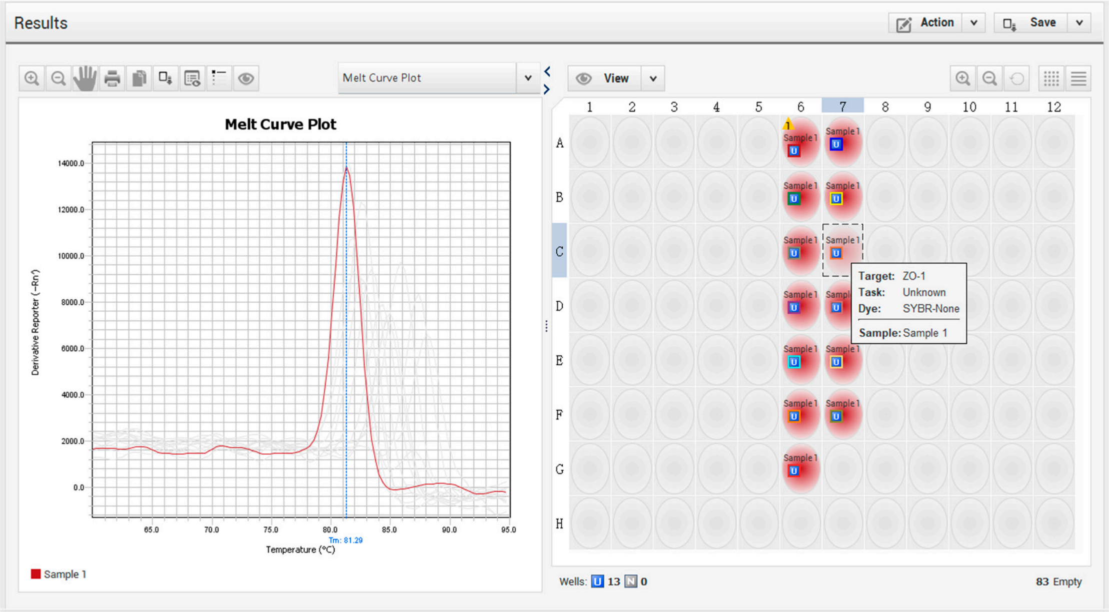
Results
Action
Save
Melt Curve Plot
View
Melt Curve Plot
Derivative Reporter (−Rn')
Temperature (°C)
Tm: 81.29
Sample 1
Target: ZO-1
Task: Unknown
Dye: SYBR-None
Sample: Sample 1
Wells: U 13 N 0
83 Empty

(D) *ZO-1*

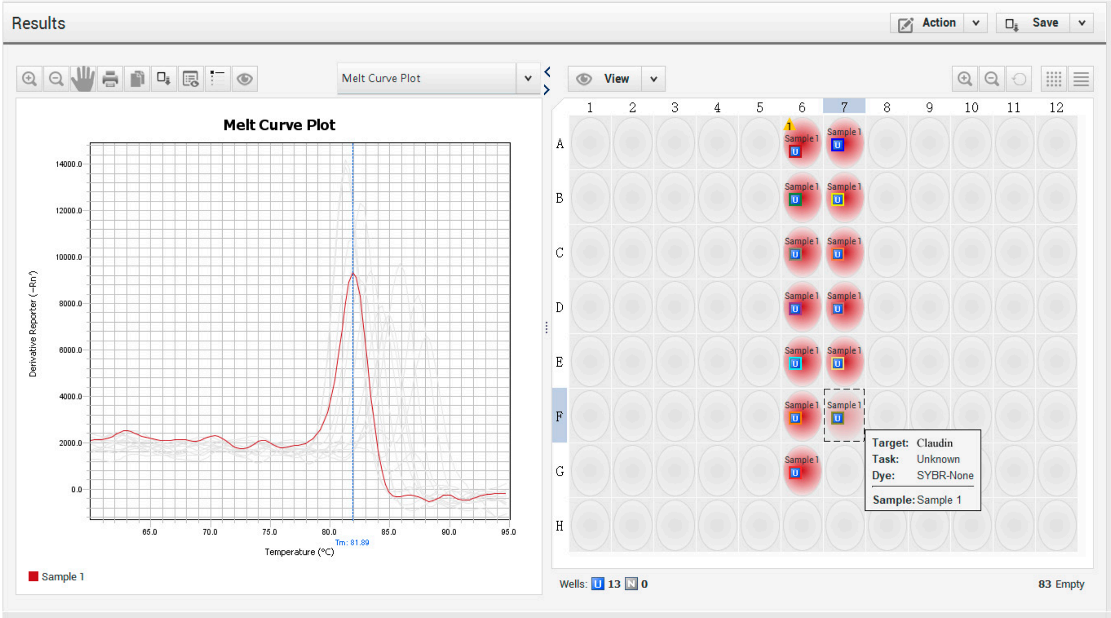

(E) *Claudin*

Figure S1. Their validation results of qPCR. (A) *β-Actin*; (B) *MUC2*; (C) *MUC4*; (D) *ZO-1*; (E) *Claudin*. *MUC2*, mucin 2; *MUC4*, mucin 4; *ZO-1*, zonula occludens-1.
